# Whole Genome Sequencing, Comparative Genome Analysis, and Biotechnological Potential of *Emericellopsis alkalina* and *E. fimetaria* (*Bionectriaceae*, *Ascomycota*) from the Sediment of Alkaline, Saline Lakes

**DOI:** 10.3390/jof12050316

**Published:** 2026-04-26

**Authors:** Vladimir V. Sokolov, Kseniya V. Malysheva, Elena N. Bilanenko, Natalia N. Markelova, Oksana A. Kolpakova, Marina L. Georgieva, Vera S. Sadykova

**Affiliations:** 1 Gause Institute of New Antibiotics, st. Bolshaya Pirogovskaya, 11, Moscow 119021, Russiamalishevaks@yandex.ru (K.V.M.); nathanmrk82@gmail.com (N.N.M.); oxkolpakova@gmail.com (O.A.K.);; 2 Faculty of Biology, Lomonosov Moscow State University, 1-12 Leninskie Gory, Moscow 119234, Russia; e_bilanenko@mail.ru

**Keywords:** *Emericellopsis*, whole-genome sequence, comparative genomics analysis, antimicrobial activity, peptaibols

## Abstract

*Emericellopsis* species from extreme environmental conditions provide a rich source of unique and biologically active secondary metabolites. The paper exhibits a comprehensive genomic analysis including complete genome sequencing, phylogenetic reconstruction, and functional annotation of two Emericellopsis species from highly saline and alkaline coastal soil ecosystems. Comparative genomics has been applied to reveal the genetic evolution, metabolic diversity, and environmental adaptation of the *Emericellopsis* genus. The genomes of *E. alkalina* E101 and *E. fimetaria* p24 have been found to encode various enzymes, including carbohydrate-active enzymes such as endoxylanases, that are useful for many ecological adaptations. The genomes of *E. alkalina* E101 and *E. fimetaria* p24 feature numerous biosynthetic gene clusters (BGCs), capable of synthesizing both known and potentially novel secondary metabolites with antimicrobial activity. Some BGCs show similarity to those producing known secondary metabolites, such as leucostatin A/B, clavaric acid, ascochlorin, (-)-mellein, and apicidin, among others. However, the majority of BGCs do not display any known similarities. Thus, comparative genomics offers new insights into the biology, adaptation, and evolutionary history of *Emericellopsis* fungi and may serve as a highly useful tool within biotechnological applications.

## 1. Introduction

The genus *Emericellopsis* (*Bionectriaceae, Hypocreales*, *Ascomycota*) was first named by Dutch botanist J.F.H. van Beyma in 1940 to accommodate the species *E. terricola* and its variety *E. terricola* var. *glabra* [1]. Fungi that belong to this genus are found within both terrestrial and marine habitats: in soil and as endophytes of plants, in animal and bird droppings, in sediments of freshwater and salt continental lakes, as epiphytes and endophytes of sea plants, in marine bottom deposits, and in the cavities of the sponges [2,3]. Currently, 31 species are recognized within the genus *Emericellopsis*, which phylogenetically is divided into two large clades—“marine” and “terrestrial” [2,3]. However, such division does not fully reflect substrate specificity since some species from the “terrestrial” clade occur in marine environments while others from the “marine” clade inhabit terrestrial ecosystems. Previously, a separate “soda” clade with only one species *E. alkalina* has been proposed adjacent to the “marine” group [4].

The species *E. alkalina* Bilanenko & Georgieva [4] was first described in 2013 from highly alkaline (pH 10.1) saline soil from the edge of soda lake Tanatar-2, Altai Krai, Russia. This species is widely distributed in coastal soils of saline and soda lakes in the Altai region and Zabaykalye (Russian Federation), later being recorded in sediment samples from saline lakes in Egypt, Red Sea sediments, and deep-sea Atlantic Ocean sediments [4,5,6,7]. The preferred substrates for this species remain debatable. It is an alkaliphilic halotolerant species, with optimal growth occurring at medium pH values ranging between 9.5 and 10.0 [4,8]. *E. alkalina* produces a complex of bioactive peptaibols known as emericellipsins (EmiA–E), which exhibit potent antifungal activity against pathogenic fungi, including drug-resistant *Candida* spp. and *Aspergillus* spp. The maximum production of these compounds occurs under high alkaline conditions, specifically at pH 10–11 [9].

The species *E. fimetaria* (Persoon) L.W. Hou, L. Cai & Crous was initially described in 1799 by the mycologist Christian Hendrik Persoon as *Leotia fimetaria* on cattle dung [10], and in 2023, based on phylogenetic reconstruction, this species was placed into the genus *Emericellopsis* within the “terrestrial” clade [2]. Most records of this species, starting with *L. fimetaria*, were listed on excrement of various animals (mainly herbivores) and birds worldwide, as noted by Lindau and Seifert, but recent findings include occurrences in other biotopes: marine sediments, sediments of the Tambukan saline lake (North Caucasus, Russia) [11,12,13,14]. It is known that strains of *E. fimetaria* produce diverse compounds with antimicrobial activity, including peptaibols like antiamoebins and terpenoid-like substances [11,12,15,16,17].

Currently, genomes of three *Emericellopsis* species have been published: *E. atlantica* [18], *E. cladophorae* [19], and *E. maritima* [20]. An unannotated genome assembly of *E. terricola* is available in GenBank (Genbank: GCA_027569275.1). The analysis of these genomes aimed at identifying clusters of genes involved in secondary metabolism (BGC) reveals their potential capacity for biosynthesis of diverse nonribosomal peptides and terpenoids, which may exhibit antimicrobial properties.

The aim of this study is whole-genome sequencing and functional annotation of two *Emericellopsis* species: *E. alkalina* E101 ex-type strain and *E. fimetaria* p24 strain, followed by an analysis of their potential as producers of different antimicrobial compounds.

## 2. Materials and Methods

### 2.1. Strains

*Emericellopsis alkalina* E101 strain (=CBS 127350 =VKM-F 4108, ex-type) was isolated from soil of the edge of soda lake Tanatar-2 in 2002 (Altai Region, Russia); soil pH 10.1, soluble salt content 73 g/kg [4]. *Emericellopsis fimetaria* p24 strain was isolated from sediment samples of the Tambukan saline lake in 2018 (Northern Caucasus, Russia); sample pH range 7.5–8.0, water mineralization 27.3 g/L [14].

All genomes utilized for comparative purposes have been obtained from publicly accessible databases: *E. atlantica* TS7 (ex-type, from the sponge *Stelletta normani*, Northern Atlantic Ocean) [18] (Genbank: GCF_019669845.1), *E. cladophorae* MUM 19.33 (=CMG 25, ex-type, endophyte of *Cladophora* sp., Atlantic Ocean) [19] (Genbank: GCF_022114955.1), *E. maritima* CBS 491.71 (ex-type, coastal water from Black Sea) [20] (Mycocosm: Ememar1) and *E. terricola* NRRL 54109 (weed from root crown crop field, USA) (Genbank: GCA_027569275.1).

Fungal strains were cultivated for 5 days in potato-glucose broth at the temperature of 25 °C and agitation speed of 120 rpm in 250 mL flask. Mycelium has been separated by centrifugation at 4740× *g* and +4 °C.

### 2.2. DNA Extraction

High-molecular-weight fungal genomic DNA has been extracted from the mycelium using the SKYHMW Plant Genomic DNA kit (SkyGen, Moskov, Russia) in accordance with the manufacturer’s instructions and used for long-read DNA sequencing (Nanopore). Genomic DNA for short-read sequencing has been obtained using a kit for the extraction and purification of genomic DNA from animal and bacterial cell cultures, animal and plant tissues (DU. Cells, tissues, blood DNA isolation kit, Biolabmix, Novosibirsk, Russia). The concentration of the extracted DNA has been estimated using UV spectrometry on an EzDrop 1000 device (Blue-Ray Biotech, New Taipei City, Taiwan), measuring the optical density of the samples at a wavelength of 260 nm. A DNA sample was considered pure if the ratio of light absorption at wavelengths of 260 nm and 280 nm (A260/280) was nearly equal to 1.8. Accurate DNA concentration determination has been performed using a Qubit 4 Fluorometer (Thermo Fisher Scientific, Waltham, MA, USA) and the Qubit dsDNA HS Assay Kit (Thermo Fisher Scientific, Waltham, MA, USA). DNA samples have been adjusted to 400 ng in a 12 µL volume and to a concentration of 30 ng/µL for subsequent library preparation for nanopore sequencing and sequencing by synthesis, respectively.

### 2.3. Library Preparation and Sequencing

The ONT (Oxford Nanopore Technologies) library has been prepared without an amplification step using the Native Barcoding kit (SQK-NBD114-24; ONT, Oxford, UK) and the NEBNext ONT Companion Module for end-repair, and ligation (New England Biolabs, Ipswich, MA, USA). Library enrichment with long fragments has been performed using a long fragment buffer (LFB) according to the manufacturer’s protocol. Sequencing has been performed for 72 h on a MinION using R10.4.1 flow cells (FLO-MIN114; ONT, Oxford, UK) and MinKNOW software (v.22.10.7, Oxford Nanopore Technologies, Oxford, UK). Base-calling has been performed using Guppy (v.6.3.8, Oxford Nanopore Technologies, Oxford, UK). The resulting ONT fast5 data has been converted to pod5 format and base-calling has been performed using Dorado Basecaller v.0.8.3 (Oxford Nanopore, UK, URL: https://github.com/nanoporetech/dorado (accessed on 21 September 2025)) in the super-precision mode (SUP), UK quality control of reads in QUAST v.5.3.0 [21].

A DNA sequence library for sequencing by synthesis has been prepared using the Raissol SG_GM Maxi reagent kit (Sesana LLC, Moscow, Russia), a system for the enzymatic preparation of genomic DNA libraries larger than 300 bp using “ShotGun Sequencing” technology. DNA fragmentation levels, library fragment size distribution, and fragment concentration have been analyzed using a Qsep400 analyzer (BiOptic, New Taipei City, Taiwan). Whole-genome sequencing has been performed using a Genoscan 4000 nucleic acid sequencer (Sesana LLC, Moscow, Russia).

Data processing has been carried out using FastQC version 0.23.1 [22], and read quality control has been done in QUAST version 5.3.0 [21]. Hybrid assembly has been conducted using Unicycler (version 0.5.1) in the mode combining long-read assembly with polishing by short reads [23].

### 2.4. Structural and Functional Annotation

For structural annotation, we employed Augustus [24] using the model species “Fusarium”, which is phylogenetically the most closely related one. After obtaining structural annotations and extracting protein sequences, we analyzed genome completeness using BUSCO (“Benchmarking Universal Single-Copy Orthologs”) in the “protein” mode with the Hypocreales odb12 orthologous gene set [25]. Additionally, we conducted orthology analysis using OrthoFinder version 3.0 [26] to detect putative pseudogenes and incorrect gene models. Functional annotation has been performed using the tools InterProScan version 6.0.0 [27,28] and eggnog-mapper (Emapper version 2) [29]. To identify carbohydrate-active enzymes, the standalone tool run_dbcan (for dbCAN3) has been applied [30,31,32]. Search for sulfatases and peptidases has been carried out against the Sulfatlas 2.3.1 [33] and MEROPS [34] databases using the BLAST tool (v. 2.12.0+) [35]. Transport proteins have been identified by searching sequence databases and HMM profiles in TCDB [36,37] using BLAST [35] and HMM-search [38]. Prediction of signal peptides has been accomplished using SignalP version 6.0 (slow-sequential mode) [39]. Filtering and data analysis have been conducted using R software (v. 4.5.3) [40].

Search for gene clusters associated with secondary metabolism (BGCs) has been carried out using the meta-server FungiSMASH (the fungal version of AntiSMASH) version 8.0.4 (https://fungismash.secondarymetabolites.org/ (accessed on 21 September 2025)) in relaxed strictness mode with additional modules enabled: KnownClusterBlast, MIBiG cluster comparison, Cluster Pfam analysis, ClusterBlast, ActiveSiteFinder, SubClusterBlast, RREFinder, and TIGRFam analysis [41].

### 2.5. Reconstruction of Phylogeny

Phylogenetic reconstruction has been performed using sequences of internal transcribed spacer regions (ITS1-5.8S-ITS2), β-tubulin (tub2), large subunit ribosomal RNA (LSU), translation elongation factor 1-alpha (tef1a), and second largest subunit of RNA polymerase II (rpb2) from type strains (Supplementary Table S1). Multiple alignment has been executed in MAFFT using the L-INS-i algorithm [42] with subsequent manual adjustments in UGENE [43]. Bayesian inference-based phylogenetic analyses have been carried out in MrBayes [44,45,46] with the most comprehensive model GTR+I+G, independent parameters for each locus, and a common tree topology. Tree visualization has been done using the treedataverse package (URL: https://github.com/YuLab-SMU/treedataverse (accessed on 21 September 2025)) in the R environment [40].

## 3. Results

A phylogenetic tree has been reconstructed based on the sequences of ITS1-5.8S-ITS2, LSU, tef1α, rpb2, and tub2 from 29 type strains of the genus *Emericellopsis*, and isolates *E. fimetaria* CBS 382.62, *E. fimetaria* p24, and *E. terricola* NRRL 54109. As an outgroup, *Stanjemonium grisellum* CBS 655.79 (ex-type) has been selected. Overall, 139 sequences have been included in the analysis (Supplementary Table S1). The search for the optimal model of nucleotide substitutions have been performed by means of IQTree (the “Model Finder” module). However, among the selected models (TIM2+F+I+G for ITS1-5.8s-ITS2, TN+F+I for LSU, TN+F+G for rpb2, tub2, and GTR+I+G for tef1α), only the model GTR+I+G has been implemented in MrBayes. We remark that this model comprises the models TIM2 and TN as particular cases.

**Figure 1:**
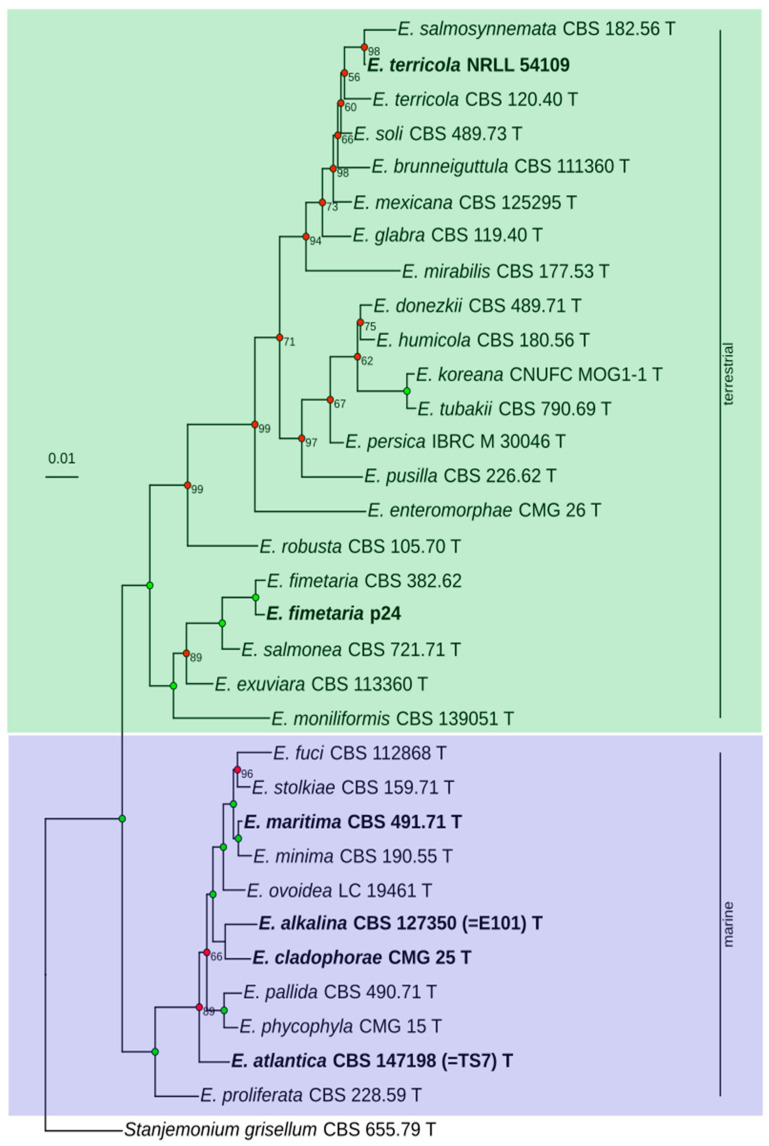
Phylogenetic tree of the *Emericellopsis* genus. Phylogeny reconstruction for ITS1-5.8s-ITS2, LSU, tef1a, rpb2, and tub2, independent parameters for each partition, GTR+I+G model. Analysis for 10,000,000 generations, burnin 25%. Bayesian posterior probability (BPP) values equal to 100% are not shown and are indicated by green dots; BPP values less than 100% are shown and indicated by red dots. T—ex-type strains. The strains whose genomes are discussed in this study are shown in bold.

*E. alkalina* E101 positions itself within the “marine” clade of the genus *Emericellopsis*, forming a distinct subclade together with *E. cladophorae* CMG 25 (Figure 1, Supplementary Figure S1). The average nucleotide identity (ANI) between the genomes of *E. alkalina* E101 and *E. cladophorae* CMG 25 is approximately 90.04%.

*E. fimetaria* p24 forms a well-supported isolated subclade within the “terrestrial” clade of the genus *Emericellopsis*, alongside *E. fimetaria* CBS 382.62, *E. salmonea* CBS 721.71 (ex-type), *E. exuviara* CBS 113360 (ex-type), and *E. moniliformis* CBS 139051 (ex-type).

The strain *E. terricola* NRRL 54109, for which genomic data is available has also been incorporated into the phylogenetic tree. This strain forms a well-supported subclade with *E. salmosynnemata* CBS 182.56 (ex-type), whereas the strain *E. terricola* CBS 120.40 (ex-type) branches externally relative to them. When adding the sequences of *E. microspora* CBS 380.62 (former type strain of the species now considered synonymous with *E. salmosynnemata*), it also forms an external group relative to the strains *E. terricola* NRRL 54109 and *E. salmosynnemata* CBS 182.56 (ex-type) (Supplementary Figure S1). This may suggest that the species identification of strain NRRL 54109 should be re-evaluated. Hereafter, we will use the species name under which the genome is currently deposited in GenBank.

### Genome Analysis

The genome of *E. alkalina* E101 (ex-type) consists of 25,722,796 base pairs assembled into 89 contigs, with an average contig size of 1,321,188 base pairs and the longest contig reaching 2,875,143 base pairs. On the other hand, the genome of *E. fimetaria* p24 consists of 24,658,382 base pairs across 43 contigs, averaging 979,341 base pairs per contig, with the longest contig measuring 3,154,750 base pairs. Detailed information can be found in Table 1.

In the genome of *E. alkalina* E101, 8612 protein-coding sequences have been predicted, with 8467 (98.3%) encoded proteins assigned to orthogroups and 8217 (95.4%) annotated at least to the level of individual domains. Similarly, in the genome of *E. fimetaria* p24, 8616 protein-coding sequences have been predicted, with 8383 (98.4%) encoded proteins classified into orthogroups and 8205 (95.2%) annotated minimally up to domain level.

For 3897 (47.4% of total annotated) genes in *E. alkalina* E101 and 3853 (46.9% of total annotated) genes in *E. fimetaria* p24, identifiers from the KEGG database have been retrieved, providing functional annotations including KOG groups. The details on the number of annotated genes, carbohydrate-active enzymes, peptidases, sulfotransferases, transport systems, and BGCs are presented in Table 2.

In the genomes of *E. alkalina* E101 and *E. fimetaria* p24, 447 and 428 genes encoding CAZymes have been respectively detected. Some transcripts contain multiple modules, typically glycoside hydrolase (GH) and carbohydrate-binding module (CBM). The most abundant CAZymes belong to families: Glycosyl-Hydrolases (GH): Family GH16 (β-1,4 and β-1,3 glucanases), Family GH18 (predominantly chitinases, secreted and intracellular), Family GH43 (β-D-xylosidases, α-L-arabinofuranosidases, and endo-α-L-arabinanases), Family GH3 (β-glucosidases), Family GH5 (mostly cellulases). Auxiliary Activity Enzymes (AA): Family AA7 (chito-oligosaccharide oxidases), Family AA3 (glucose dehydrogenases), Family AA9 (cellulose mono-oxygenases). Glycosyl Transferases (GT): Family GT2 (chitin synthases). Additionally, a significant number of enzymes from family CE5 (acetylesterases) have been observed. The genome of the obligately alkaliphilic fungus *Sodiomyces alkalinus* CBS 110278 [47], which is found in the same saline alkaline lakes as *Emericellopsis alkalina* [48], contains significantly fewer CAZymes from all classes except for AA. Also, the total number of transporter proteins in *S. alkalinus* is lower (Supplementary Table S2).

In the genome of *E. alkalina* E101, 73 carbohydrate-active enzymes possess sulfatase domains, primarily corresponding to glycosyl-hydrolases with β-glucosidase activity. There is also a single copy of a gene coding for α-L-fucosidase (EC 3.2.1.51), suggesting that *E. alkalina* E101 potentially degrades fucoidans that are present in brown algae cell walls and other sulfated polysaccharides. In the genome of *E. fimetaria* p24, 53 carbohydrate-active enzymes feature sulfatase domains, similarly containing a gene for α-L-fucosidase (EC 3.2.1.51).

In the genomes of *E. alkalina* E101 and *E. fimetaria* p24, 2444 and 2324 transport proteins, respectively, have been annotated across all major classes, including channels/pores, electrochemical potential-driven transporters, primary active transporters, group translocators, transmembrane electron carriers, accessory factors involved in transport, and incompletely characterized transport systems. Secondary Metabolite Biosynthetic Gene Clusters (BGCs): in the genome of *E. alkalina* E101, 36 BGCs have been predicted, consisting of: 10 Terpene, 9 Non-Ribosomal Peptide Synthase (NRPS), 5 NRPS-like, 5 Polyketide Synthase (PKS): 4 Type I PKS (T1PKS), 1 Type III PKS (T3PKS), 7 Hybrid NRPS+T1PKS clusters. Some BGCs show similarity to those producing known secondary metabolites, such as leucostatin A/B, clavaric acid, ascochlorin, (-)-mellein, apicidin, among others. However, the majority of BGCs do not display any known similarities. In the genome of *E. fimetaria* p24, 46 BGCs have been discovered, comprising: 14 Terpene, 12 Non-Ribosomal Peptide Synthase (NRPS), 6 NRPS-like, 5 Polyketide Synthase (PKS), 4 Type I PKS (T1PKS), 1 Type III PKS (T3PKS), 7 Hybrid NRPS+T1PKS clusters. Several BGCs demonstrate similarity to those responsible for known secondary metabolites, including ascochlorin, helvolic acid, emerimicin, cytochalazines, etc. However, the majority of BGCs lack any known similarity. The whole genome sequence of the strains has been deposited in the NCBI database under the following accession numbers PRJNA1374271 for *E. fimetaria* p24 and PRJNA1374265 for *E. alkalina* E101.

The analysis of the genomes of *E. alkalina* E101 and *E. fimetaria* p24 using the BUSCO database (Hypocreales odb12, protein mode) indicates sufficient genome completeness: the proportion of single-copy genes is 96.4% for *E. alkalina* E101 and 96.1% for *E. fimetaria* p24. This result is quite good considering the absence of RNA-seq data and the use of a non-optimal model for Augustus.

**Figure 2:**
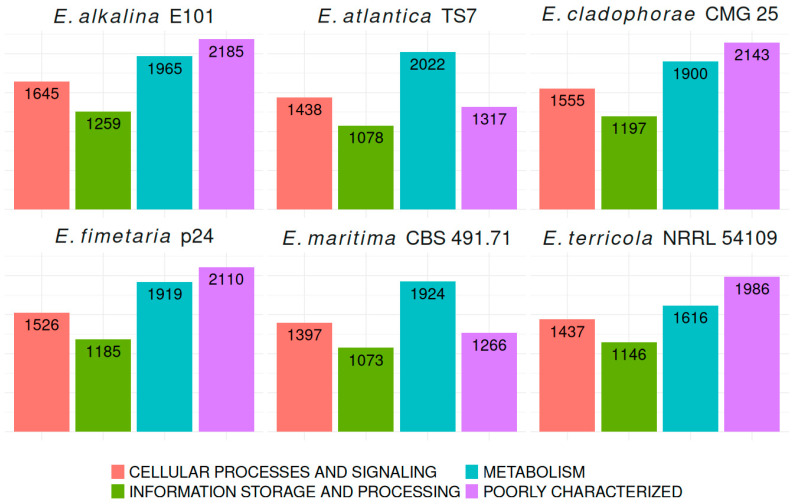
Comparative KOG features for *Emericellopsis* genomes.

To compare the functional annotation across different species within the genus *Emericellopsis*, we present the number of annotated genes categorized into respective KOG groups for four genomes: *E. atlantica* TS7, *E. maritima* CBS 491.71, *E. cladophorae* MUM 19.33, and *E. terricola* NRRL 54109 (Figure 2). These comparisons allow one to understand similarities and differences in gene function distribution among closely related fungal species.

**Figure 3:**
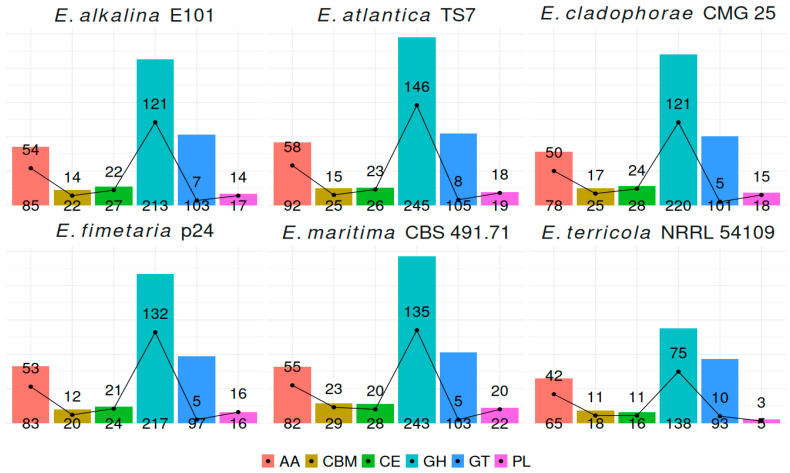
Gene clusters encoding carbohydrate-active enzymes (CAZymes) in *Emericellopsis* genomes (bars) and CAZymes with signal peptide (black line). AA—Auxiliary activity, CBM—carbohydrate-binding module, CE—carbohydrate esterase, GH—glycosyl hydrolase, GT—glycosyl transferase, PL—polysaccharide lyase.

To ensure comparable results of enzyme annotation involved in carbohydrate metabolism, transport proteins, and biosynthetic gene clusters (BGCs), six genomes of *Emericellopsis* species: *E. alkalina* E101, *E. fimetaria* p24, *E. terricola* NRRL 54109, *E. cladophorae* CMG 25, *E. atlantica* TS7, and *E. maritima* CBS 491.71 have been analyzed. Figure 3 illustrates the overall distribution of carbohydrate-metabolizing enzymes and the proportion of secreted enzymes among the studied *Emericellopsis* species. It highlights considerable inter-species differences reflecting diverse ecological niches and trophic preferences of these fungi.

**Figure 4:**
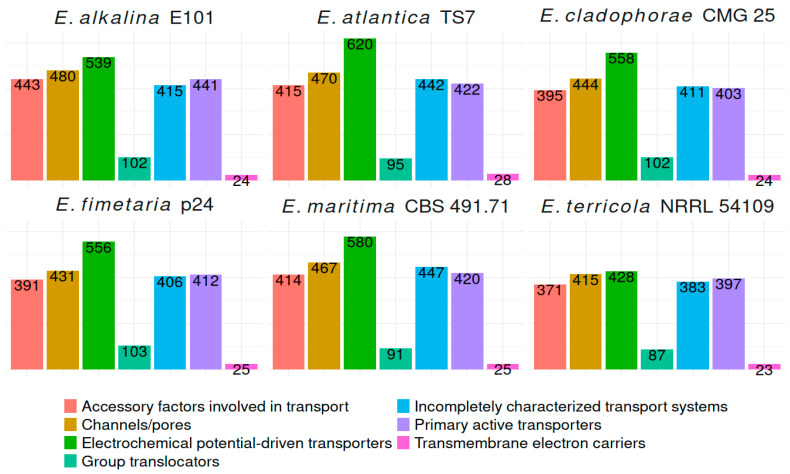
Transporter classes in *Emericellopsis* genomes.

A summary of the quantity of transport proteins belonging to different classes in the analyzed *Emericellopsis* genomes is presented in Figure 4.

**Figure 5:**
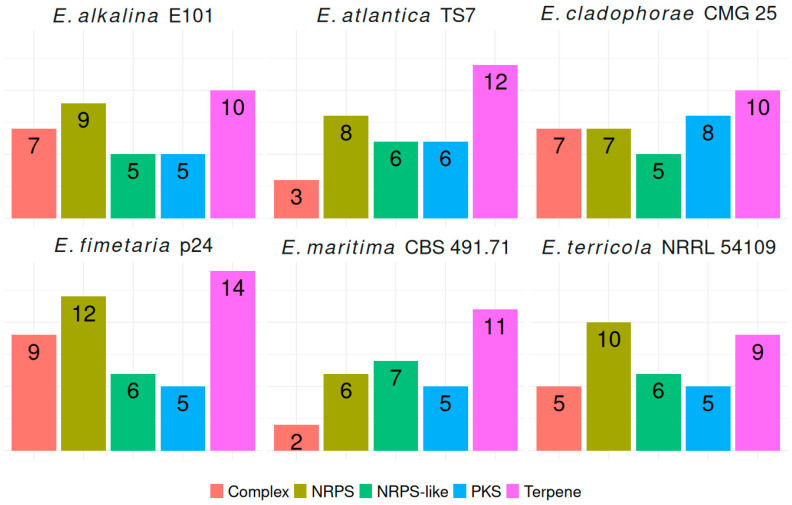
BGC in *Emericellopsis* genomes. Complex—complex BGC, NRPS—non-rybosomal peptide synthase, NRPS-like non-ribosomal peptide synthase like, PKS—polyketide synthase, Terpene—terpene synthase.

The analyzed genomes of *Emericellopsis* species differed little in the number of predicted BGCs (Figure 5).

Only several BCGs showed homologies with known clusters, which are putatively responsible for producing compounds such as a leucinostatin, ascochlorin, emerimicins, clavaric acid.

**Figure 6:**
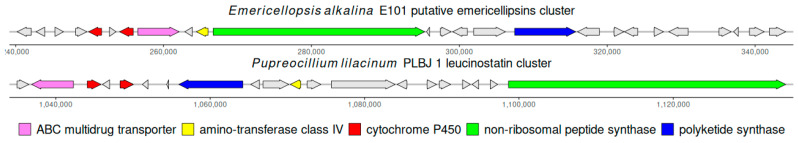
Comparison of the BGC of leucinostatin from *Purpureocillium lilacinum* PLBJ 1 and the putative BGC of emericellipsins from *Emericellopsis alkalina* E101.

Based on antiSMASH prediction, we found that *Emericellopsis alkalina* E101 contains one predicted cluster showing similarity to the leucinostatin cluster from *Purpureocillium lilacinum* (Figure 6), and we suggest that this BGC is responsible for the synthesis of emericellipsins, nonribosomal peptides belonging to the peptaibol group.

Importantly, the BGCs discovered in the genomes of *E. alkalina* E101 and *E. fimetaria* p24 encompass all genes necessary for ascochlorin synthesis (Supplementary Figure S2). Two biosynthetic gene clusters (BGCs) resembling those associated with emerimicins from *E. tubakii* PF have been identified in the genome of *E. fimetaria* p24 (Supplementary Figure S3).

The strain *E. fimetaria* p24 exhibits strong experimental antimicrobial activity against Gram-positive and Gram-negative bacteria as well as mould fungi (Supplementary Table S3). These could be established molecules like ascochlorin or helvolic acid, whose corresponding biosynthetic pathways have been identified within its genome, or novel metabolites originating from other biosynthetic gene clusters without any database homologues. Meanwhile, another strain, *E. alkalina* E101, is capable of producing emericellipsins—a group of peptaibols possessing strong antifungal properties [49,50]. Considering their proven antimicrobial potential combined with extensive biosynthetic capacity encoded by numerous gene clusters in their genomes, these two strains warrant further investigation to explore their potential as sources of new antimicrobial compounds.

## 4. Discussion

Genomics plays an important role in understanding the antimicrobial activity, biotechnological potential, and ecological roles of fungi. At present, genomic resources for the genus *Emericellopsis* are scarce, and genome information is available in NCBI for only four species. In this study, the whole genome of two more species *E. alkalina* and *E. fimetaria* has been sequenced and assembled. There were no significant differences in genome size, GC content, gene cluster type and number compared with other fungi of the same genus that had been reported. We have annotated and analyzed the genes encoding proteins involved with substrate specificity of fungi. Overall, the number of CAZymes in the genomes of *Emericellopsis* shows minor variations among different species, with few exceptions. The sequencing coverage is quite low; however, the results of genome completeness assessment allow us to consider these assemblies suitable for functional annotation and subsequent cautious interpretation of its outcomes.

The genome of the obligate alkaliphilic fungus *Sodiomyces alkalinus* CBS 110278, inhabiting in similar saline alkaline lakes to those where *E. alkalina* E101, exhibits a considerably lower number of CAZymes with the exception of class AA enzymes. It has been shown that *S. alkalinus* employs feeding strategies distinct from those of *E. alkalina* within the same biotope [48,51,52]. Adaptation strategies to extreme conditions such as high values pH and high concentrations of Na+ ions for both *E. alkalina* and *S. alkalinus* may be similar. In comparison with other species of *Emericellopsis*, *E. alkalina* exhibits an increased number of transporter proteins associated with adaptation to high pH and sodium ion concentration, comparable to their amount in *S. alkalinus* (Supplementary Table S2).

The variety of glycosyl-hydrolase enzymes with different substrate specificities in the genomes of *E. alkalina* E101 and *E. fimetaria* p24, along with a large number of enzymes presumably hydrolyzing plant cell wall polysaccharides (cellulose, xylan, pectin), suggests a typically saprotrophic lifestyle for these species. In the case of *E. fimetaria*, this assumption generally aligns with the substrate preference of the species. However, for *E. alkalina* E101, one would expect a greater utilization of chitin as a carbon source, given that typical habitats of this species are heavily populated by brine shrimp *Artemia salina* [53]. Nevertheless, no increase in the number of chitinase-encoding genes is observed in its genome. To determine whether *Emericellopsis alkalina* E101 can utilize alternative carbon sources such as chitin and sulfated polysaccharides from unicellular algae, additional experiments are needed aimed at assessing enzymatic activity towards these substrates, along with RNA-seq data to evaluate expression levels of corresponding enzymes under different conditions. In the genome of *E. alkalina* E101, there is no increase observed in the number of known fungal “pH sensors,” specifically PalH/Rim21 [54], compared to other species within the genus *Emericellopsis*. However, an increased number of Na+/H+ antiporters is noted, including homologs of yeast vacuolar/endosomal sodium tolerance protein NHX1, which plays a critical role in tolerating high concentrations of Na+ [55]. Additionally, several other electrochemical potential-driven transporters (EPT) are present. These EPT proteins play crucial roles in adaptation to environmental conditions. Specifically, Na+/H+ antiporters and P-type ATPases facilitate removal of sodium ions from cells [56], while H+-ATPases and V-ATPases regulate intracellular pH [57]. Furthermore, the genome contains an elevated number of genes encoding proteins homologous to SOS2 (Salt Overly Sensitive-2) from *Arabidopsis thaliana*, where SOS2 activates Na+/H+ antiporter function in response to excess Na+ ions [58]. The experimental characterization of many EPT functions remains incomplete, limiting conclusions about mechanisms underlying halotolerance and pH tolerance in microfungi like *E. alkalina*. This lack of detailed information necessitates further research to fully understand how this fungus adapts to changes in external Na+ concentration and pH.

The slightly larger number of BGCs in the genome of *E. fimetaria* p24 compared to other species may represent an adaptive strategy to its characteristic habitat—the dung of herbivorous animals. This nutrient-rich environment hosts a diverse microbial community, leading to intense competition for nutrients. Consequently, a higher production of antimicrobial secondary metabolites could confer a competitive advantage to *E. fimetaria*. Experimental evidence supports this hypothesis. Studies conducted by Singh and Webster revealed that *E. fimetaria* (originally referred to as *Stilbella*) inhibits the growth of coprophilic fungi and bacteria both in culture and in substrates. They demonstrated that antagonistic interactions occur primarily through the release of substances with antimicrobial and antifungal properties rather than direct hyphal contact [59]. Thus, the enhanced capacity for producing secondary metabolites likely enhances the fitness of *E. fimetaria* in its ecologically challenging habitat.

One of the proposed biosynthetic gene clusters (BGCs) in the genome of *E. alkalina* E101 is hypothesized to encode the biosynthesis pathway for emericellipsins—a class of peptaibols exhibiting potent antifungal and cytotoxic activities (Figure 6). Emericellipsins A-E consist of nine amino acids, including uncommon residues such as 2-amino-6-hydroxy-4-methyl-8-oxodecanoic acid (AHMOD) and β-alanine [9,60,61]. Leucinostatins, another class of nonribosomal lipopeptides structurally similar to peptaibols, incorporate rare components like AHMOD. Their biosynthesis requires polyketide synthase, aminotransferase, and cytochrome-P450 enzymes [62].

Both *E. alkalina* E101 and *E. fimetaria* p24 BGCs resemble those associated with ascochlorin (ilicicolin D) biosynthesis. Ascochlorin, a terpenoid compound, has originally been known for their antibacterial, antifungal, and cytotoxic properties [63,64,65]. Iliciolins, including ascochlorin (ilicicolin D), exert their effects by inhibiting fungal cytochrome bc1-reductase [66], making them attractive candidates for developing novel antifungal agents. The biosynthetic pathway of ascochlorin via orsellinic acid, as described in *Acremonium egyptiacum*, involves several key enzymes: polyketide synthase, prenyltransferase, reductase, halogenase, epoxidase, terpene cyclase, and dehydrogenase. Moreover, a transcription-regulating protein is also present within the BGC. Intermediate products in this pathway include ilicicolins A, B, and C, which themselves exhibit antimicrobial activity [67]. Importantly, the BGCs discovered in the genomes of *E. alkalina* E101 and *E. fimetaria* p24 encompass all genes necessary for ascochlorin synthesis.

We have found that two BGCs of *E. fimetaria* p24 exhibit similarity to the BGC of emerimicins from *E. tubakii* [68]. One of the detected BGCs includes two nonribosomal peptide synthetases (NRPSs): one with 15 domains and an acyltransferase domain, another with only two domains. The second BGC comprises an NRPS with 16 domains and an acyltransferase domain. Based on this observation, we propose that one BGC produces peptaibols antiamoebins consisting of 16 amino acid residues, previously reported in various strains of *E. fimetaria* [18]. Emerimicins belong to the family of nonribosomal peptide antibiotics, and possess broad-spectrum anti-microbial activity against Gram-positive bacteria, fungi, and protozoans [69,70]. These compounds consist of either 15 (all except for IIA and IIB) or 16 amino acid residues. The second BGC, featuring two NRPSs, might produce either. Furthermore, *E. fimetaria* p24 produces several compounds exhibiting antimicrobial activity against both Gram-positive and Gram-negative bacteria, as well as fungi [71].

BGCs that do not match any known clusters may either provide biosynthesis pathways for novel secondary metabolites or remain inactive or inducible only under specific conditions. Based solely on genomic BGC prediction results, we cannot draw definitive conclusions regarding their functions. No genes have been identified in predicted BGCs that could be unequivocally interpreted as responsible for self-resistance against their possible products. This might be due to limitations in available databases and annotation algorithms, as well as alternative producer tolerance mechanisms.

## 5. Conclusions

This study presents the genomes of *E. alkalina* E101 (ex-type) and *E. fimetaria* p24, isolated from extreme biotopes like saline alkaline lake sediments characterized by high pH and NaCl concentration. Overall, although the precise mechanisms that allow these fungi to thrive in their natural biotopes remain unclear, the set of enzymes involved in carbohydrate degradation points towards generalist strategies promoting colonization of diverse ecological niches. Similar profiles of transport proteins, particularly those from the group of electrochemical potential-driven transporters, may indicate similar adaptation mechanisms of *Emericellopsis* species to elevated concentrations of NaCl.

Clearly, the conclusions about substrate specificity and adaptation mechanisms to extreme conditions cannot be made solely based on genome comparison data, and further research is necessary. The abundant presence of poorly studied biosynthetic gene clusters (BGCs) in the genomes of *E. alkalina* E101 and *E. fimetaria* p24 might suggest their biotechnological potential. Further studies of these fungi will advance our understanding of the evolution and ecology of extremotolerant fungi, while exploring BGCs and their possible products could lead to the discovery of new secondary metabolites with antimicrobial activity.

## Figures and Tables

**Table 1:** Assembly metrics for *E. alkalina* E101 and *E. fimetaria* p24 genomes.

Assembly Metric	*E. alkalina* E101	*E. fimetaria* P24
Total length (bp)	25,722,796	24,658,382
Coverage	<10x	<10x
BUSCO score ^1^	96.4%	96.1%
Contigs	89	43
GC (%)	54.89	53.70
N50 (bp)	1,321,188	979,341
L50 (bp)	8	9

^1^ protein mode, % of complete single-copy genes form Hypocreales_odb12 database.

**Table 2:** Annotation features for *E. alkalina* E101 and *E. fimetaria* p24 genomes.

Annotation Features	*E. alkalina* E101	*E. fimetaria* P24
Genes (total)	8612	8616
Genes in orthogroups	8467	8383
CDS	8612	8616
Annotated genes	8217	8205
Orthologues in KEGG	3897	3853
CAZymes	447	428
Peptidases	454	442
Sulfatases	469	450
Transporters	2444	2324
BGC (total)	36	46
BGC, terpenes	10	14
BGC, NRPS	9	12
BGC, NRPS-like	5	6
BGC, PKS	5	5
BGC, complex ^1^	7	9

^1^ NRPS+PKS in peptaibols BGCs.

## Data Availability

All sequence data are available in NCBI GenBank following the accession numbers in the manuscript.

## Supplementary Materials

The following supporting information can be downloaded at: https://www.mdpi.com/article/10.3390/jof12050316/s1, Figure S1: Phylogenetic tree of the *Emericellopsis* genus included *E. microspora* CBS 380.62 (syn. *E. salmosynnemata*); Table S1: Genbank accession numbers for sequences used in the phylogenetic analysis fungi of the *Emericellopsis* genus; Table S2: Comparison for *E. alkalina* CBS 127350 (=E101) and *S. alkalinus* CBS 110278 genomes; Table S3: Antimicrobial activity of *E. fimetaria* p24; Figure S2: Comparison of BGC of ascochlorin from *Acremonium egyptiacum* F-1392, *E. alkalina* E101 and *E. fimetaria* p24; Figure S3: Comparison of BGCs from the *E. fimetaria* p24 genome similar to the BGC of emerimicins from the *E. tubakii* PF genome.
