# Supplementary material for: Whole Genome Sequencing, Comparative Genome Analysis, and Biotechnological Potential of Emericellopsis alkalina and E. fimetaria (Bionectriaceae, Ascomycota) from the Sediment of Alkaline, Saline Lakes
Source: J Fungi (Basel). 2026 Apr 26;12(5):316. doi: 10.3390/jof12050316 (PMC13207871; doi:10.3390/jof12050316)
Supplement: Supplementary file 1 [file jof-12-00316-s001.zip › jof-4193291-supplementary.pdf]

Supplementary materials

Table S1. Genbank accession numbers for sequences used in the phylogenetic analysis fungi of the *Emericellopsis* genus.

Species	Strains	Genbank accession numbers for sequences				
		**ITS**	**tub2**	**rpb2**	**tef1a**	**LSU**
E. alkalina Bilanenko & Georgieva, 2013	CBS 127350 T	KC987171	KC987133	OQ453930.1	OQ470836.1	OQ055455
E. atlantica L.W. Hou, Crous, Rämä & Hagestad 2021	CBS 147198 T	OL539742.1	OL634966	OL634963.1	OL634964.1	PV272965
E. brunneiguttula L.W. Hou, L. Cai & Crous 2023	CBS 111360 T	AY632654.1	AY632689	OQ453932.1	OQ470838.1	OQ055457
E. cladophorae M. Gonçalves, T. Vicente & A. Alves, 2019	MUM 19.33 T	MK986711.1	MK984311	genome[1]	genome	genome
E. donezkii Beliakova, 1974	CBS 489.71 T	NR_156195.1	AY632674	OQ560700.1	OQ470839.1	OQ055458
E.enteromorphae M. Gonçalves, T. Vicente & A. Alves, 2019	MUM 19.34 T	MK986712	AY632674.1	–[2]	–	–
E. exuviara E. exuviara (Sigler, Zuccaro, Summerbell & Paré) L.W. Hou, L. Cai & Crous, 2023	CBS 113360 T	AY882946	AY882947	OQ453933.1	OQ470840.1	OQ055459
E. fimetaria (Pers.) L.W. Hou, L. Cai & Crous, 2023	CBS 382.62	AY632666	AY632682	OQ453939.1	OQ470846.1	OQ055465.1
E. fimetaria (Pers.) L.W. Hou, L. Cai & Crous, 2023	p24	genome	genome	genome	genome	genome
E.fuci (Summerbell, Zuccaro & W. Gams) L.W. Hou, L. Cai & Crous, 2023	CBS 112868 T	AY632653	AY632690.1	OQ453957.1	OQ470864.1	OQ055471
E. glabra (J.F.H. Beyma) Backus & Orpurt , 1962	CBS 119.40 T	NR_145024.1	AY632673.1	OQ453965.1	OQ470872.1	MH867552
E. humicola (Cain) Cain ex Grosklags & Swift, 1957	CBS 180.56 T	NR_145025.1	AY632675.1	OQ453966.1	OQ470873.1	OQ055479
E. koreana Hyang B. Lee, S.J. Jeon & T.T.T. Nguyen, 2019	CNUFC MOG1-1 T	MH173304	MH243035	–	–	–
E. maritima Beliakova, 1970	CBS 491.71 T	KC987175	KC987137	KC999033.1	OQ470874.1	OQ055480
E. mexicana Lin Zhao & Crous, 2025	CBS 125295 T	PV272756.1	–	PV273335.1	PV273536.1	PV272977

E. minima Stolk, 1955	CBS 190.55 T	KC987173	KC987135	KC999031.1	OQ470876.1	OQ055482
E. mirabilis (Malan) Stolk, 1955	CBS 177.53 T	AY632656	–	OQ453969.1	OQ470878.1	OQ055484
E.moniliformis (A. Giraldo, Deanna A. Sutton & Guarro) L.W. Hou, L. Cai & Crous, 2023	CBS 139051 T	LN810516	LN810523	OQ453972.1	OQ470881.1	OQ055486
E. ovoidea F. Liu, S. Song & L. Cai, 2024	LC19461 T	PP357003.1	–	PP437538.1	PP437536.1	PP392797.1
E. pallida Beliakova, 1974	CBS 490.71 T	KC987176	KC987138	KC999034.1	KC998998.1	OQ055487
E. persica Papizadeh, Wijayaw, Soudi & K.D. Hyde, 2016	IBRC-M 30046 T	KX668543	–	–	–	–
E.phycophila M. Gonçalves, T. Vicente & A. Alves, 2019	MUM 19.32 T	MK986701	MK984301	–	–	–
E. proliferata Lin Zhao & Crous, 2025	CBS 228.59 T	PV272742.1	–	PV273321.1	PV273522.1	PV272964
E. pusilla P.N. Mathur, Sukapure & Thirumalachar, 1962	CBS 226.62 T	MH858143	–	OQ453974.1	OQ470884.1	OQ055489
E. robusta Emden & W. Gams, 1971	CBS 105.70 T	OQ429577	–	OQ453975	OQ470885	OQ055490
E. salmonea (W. Gams & Lodha) L.W. Hou, L. Cai & Crous, 2023	CBS 721.71 T	MH860309	–	OQ453976.1	OQ470886.1	OQ055491
E. salmosynnemata Grosklags & Swift, 1957	CBS 182.56 T	MH857571.1	–	OQ453977.1	OQ470887.1	OQ055492
E. soli Lin Zhao & Crous, 2025	CBS 489.73 T	PV272765.1	–	PV273343.1	PV273544.1	PV272986
E. stolkiae D.E. Davidson & M. Christensen, 1971	CBS 159.71 T	NR_156197.1	AY632684.1	OQ453978.1	OQ470888.1	OQ055493
E. terricola J.F.H. Beyma, 1940	CBS 120.40 T	U57676	–	OQ453980.1	OQ470890.1	OQ055495
E. terricola J.F.H. Beyma, 1940	NRRL 5409 T	genome	genome	genome	genome	genome
E. tubakii (Gams) L.W. Hou, L. Cai & Crous, 2023	CBS 790.69 T	MH859429	–	OQ453983.1	OQ470893.1	OQ055498
S. grisellum W. Gams, Schroers & M. Chr., 1998	CBS 655.79 T	NR_156501.1	AY632687.1	OQ454267.1	OQ471199.1	OQ430117

[1] sequences from genome by BLAST

[2] sequences not available

T – ex-type strains

Table S2. Comparison for *E. alkalina* CBS 127350 (=E101) and *S. alkalinus* CBS 110278 genomes.

Annotation features	*E. alkalina* CBS 127350(=E101)	*S. alkalinus* CBS 110278
CAZymes	447	363
AA	54	79
GH	213	150
GT	103	89
PL	17	6
CE	27	23
CBM	22	16
Transporters	2444	2005
3.A.2.2[1]	14	14
3.A.3.8[1]	5	4
2.A.36[1]	8	3
2.A.37[1]	2	2
2.A.38[1]	5	4
2.A.1.9[1]	2	1
2.A.20[1]	5	4

[1] families important for adaptation to high pH and Na+ concentrations, classification according to tcdb.org

Table S3. Antimicrobial activity of *E. fimetaria* p24 [71]

Antimicrobial activity[1]	*E. fimetaria* p24
Aspergillus niger INA00760	9 – 13[2]
Candida albicans ATCC 2091	0 – 13[2]
Escherichia coli ATCC 25922	0 – 17[2]
Staphylococcus aureus ATCC 29213	17 – 19[2]

[1] mm of growth inhibition zone by by disc-diffusion method

[2] depending on the carbon source

The antimicrobial activity was influenced by the carbon source in the culture medium.

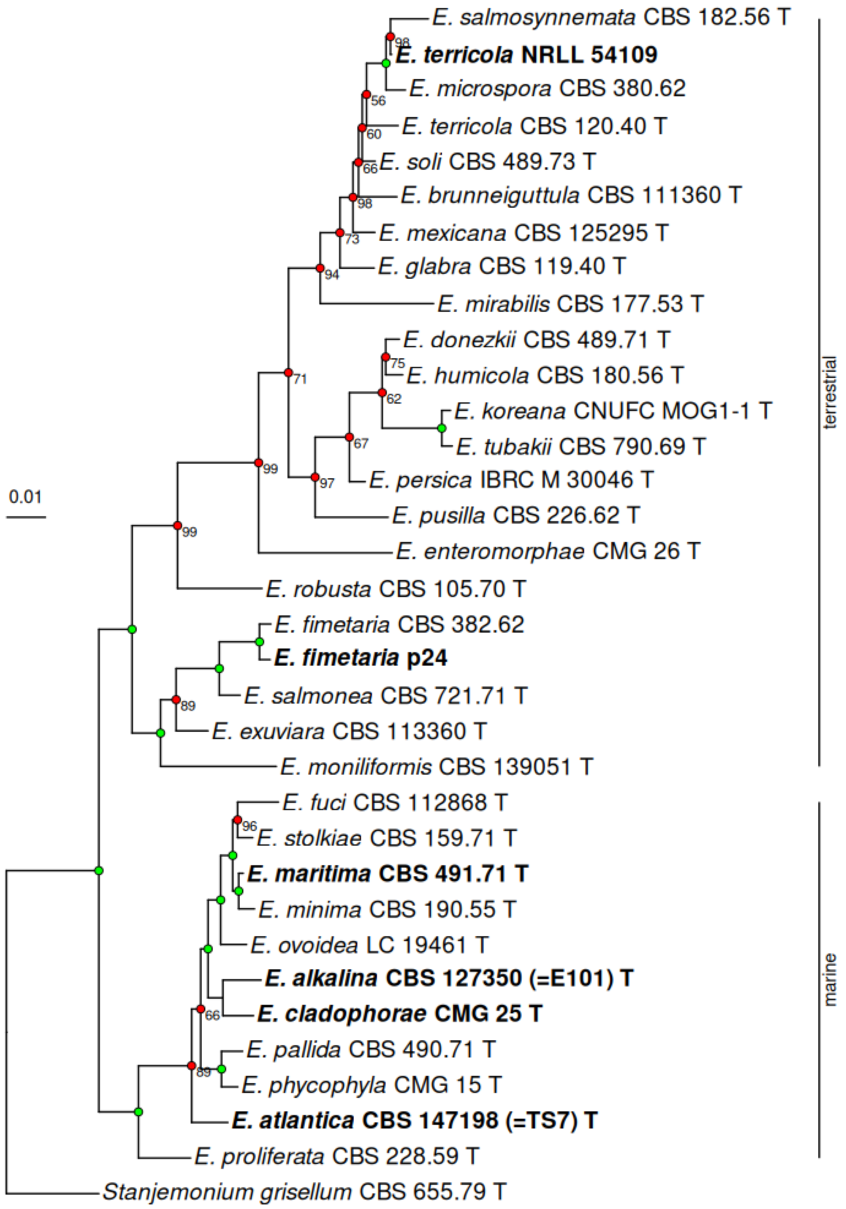

Figure S1. Phylogenetic tree of the *Emericellopsis* genus included *E. microspora* CBS 380.62 (syn. *E. salmosynnemata*). Phylogeny reconstruction for ITS1-5.8s-ITS2, LSU, tef1a, rpb2, and tub2, independent parameters for each partition, GTR+I+G model. Analysis for 10,000,000 generations, burnin 25%. Bayesian posterior probability (BPP) values equal to 100% are not shown and are indicated by green dots; BPP values less than 100% are shown and indicated by red dots. T - ex-type strains. Strains whose genomes are discussed in this study are shown in bold.

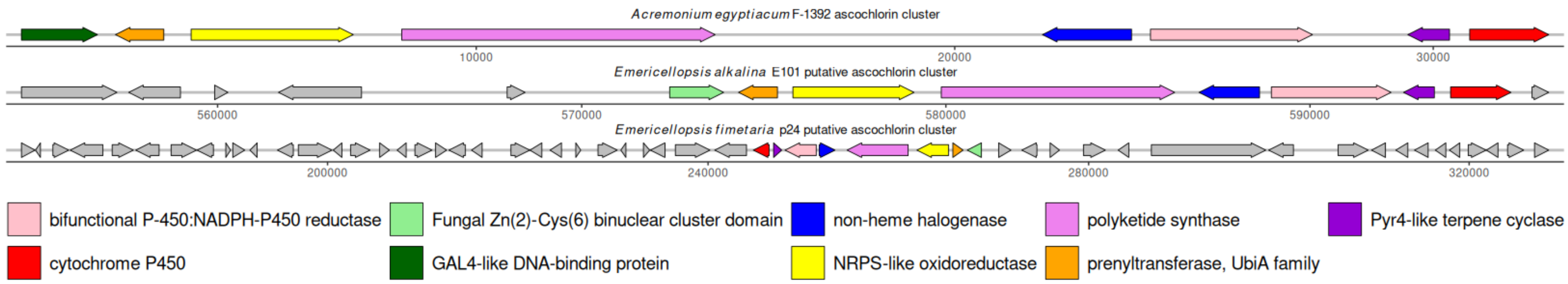

Figure S2. Comparison of BGC of ascochlorin from *Acremonium egyptiacum* F-1392, *Emericellopsis alkalina* E101 and *Emericellopsis fimetaria* p24.

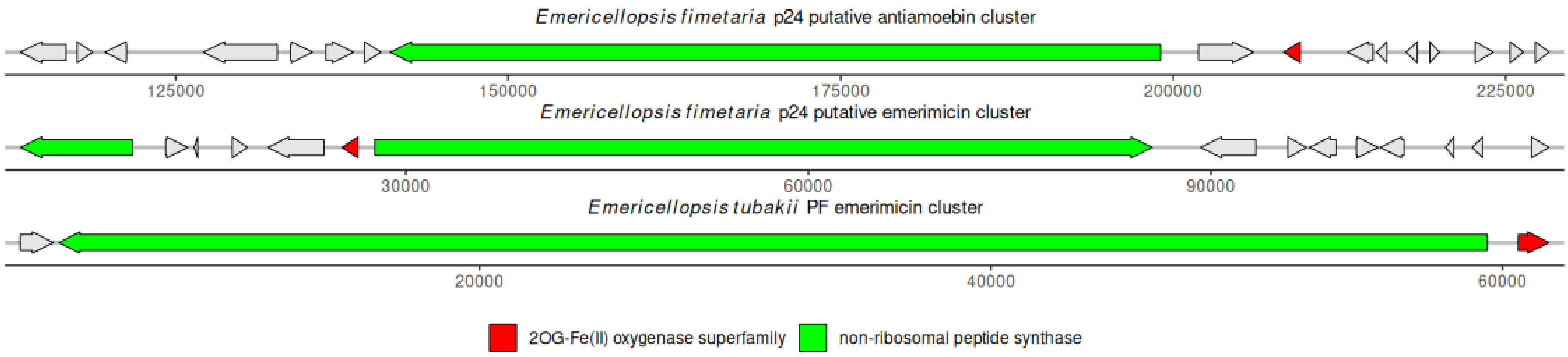

Figure S3. Comparison of BGCs from the *E. fimetaria* p24 genome similar to the BGC of emerimicins from the *E. tubakii* PF genome.
